# Clinical application of circulating tumor cells

**DOI:** 10.1515/medgen-2023-2056

**Published:** 2023-12-05

**Authors:** Nikolas H. Stoecklein, Julia Oles, Andre Franken, Hans Neubauer, Leon W.M.M. Terstappen, Rui P.L. Neves

**Affiliations:** 1 Heinrich-Heine University Düsseldorf General, Visceral and Pediatric Surgery University Hospital and Medical Faculty Düsseldorf Deutschland; 2 Heinrich-Heine University Düsseldorf General, Visceral and Pediatric Surgery University Hospital and Medical Faculty Düsseldorf Deutschland; 3 University Hospital and Medical Faculty of the Heinrich-Heine University Düsseldorf Department of Obstetrics and Gynecology Düsseldorf Deutschland; 4 University Hospital and Medical Faculty of the Heinrich-Heine University Düsseldorf Department of Obstetrics and Gynecology Düsseldorf Deutschland; 5 Heinrich-Heine University Düsseldorf General, Visceral and Pediatric Surgery University Hospital and Medical Faculty Düsseldorf Deutschland; 6 Heinrich-Heine University Düsseldorf General, Visceral and Pediatric Surgery University Hospital and Medical Faculty Düsseldorf Deutschland

**Keywords:** Circulating Tumor Cells, CTCs, clinical relevance, prediction, CSF

## Abstract

This narrative review aims to provide a comprehensive overview of the current state of circulating tumor cell (CTC) analysis and its clinical significance in patients with epithelial cancers. The review explores the advancements in CTC detection methods, their clinical applications, and the challenges that lie ahead. By examining the important research findings in this field, this review offers the reader a solid foundation to understand the evolving landscape of CTC analysis and its potential implications for clinical practice. The comprehensive analysis of CTCs provides valuable insights into tumor biology, treatment response, minimal residual disease detection, and prognostic evaluation. Furthermore, the review highlights the potential of CTCs as a non-invasive biomarker for personalized medicine and the monitoring of treatment efficacy. Despite the progress made in CTC research, several challenges such as standardization, validation, and integration into routine clinical practice remain. The review concludes by discussing future directions and the potential impact of CTC analysis on improving patient outcomes and guiding therapeutic decision-making in epithelial cancers.

## Introduction

Circulating tumor cells (CTCs) have long been recognized as cancer cells originating from solid malignant tumors that have gained access to the bloodstream and can be detected in peripheral blood samples. The first documented description of CTCs is credited to Australian physician Thomas Ashworth, dating back to 1869 [1]. Ashworth made this remarkable observation when he analyzed a venous blood sample taken from a deceased patient with multiple subcutaneous tumors. He noted the presence of cells in the blood that displayed morphological similarities to the cancer cells found within the tumors. Ashworth hypothesized that these cells in the bloodstream might be associated with the spread and multiplication of tumors. He wrote that ‘The fact of cells identical with those of the cancer itself being seen in the blood may tend to throw some light upon the mode of origin of multiple tumors existing in the same person.’ and formulated a concept which was way ahead of its time. However, his findings, unfortunately, faded into obscurity over the years. The systematic investigation of CTCs began between the 1930s to 1960s, coinciding with the establishment of the concept of hematogenous metastasis [2]. During this period, researchers primarily focused on exploring whether CTCs could serve as an indicator of metastatic risk in patients with localized cancers who were undergoing surgical interventions. However, effective methods for enriching CTCs were limited, and their detection relied mostly on conventional cytopathological techniques. Consequently, detecting CTCs posed significant challenges and yielded results that, by today’s standards, were somewhat unreliable. Since then, the field of liquid biopsy analysis has experienced a transformative shift, primarily driven by remarkable advancements in molecular biology and genetics. These advancements have paved the way for exciting possibilities and expanded applications of CTC analysis. From a contemporary perspective, CTCs have garnered significant interest due to their unique ability to provide direct access to systemic cancer at all stages of its development, effectively offering a “real-time liquid biopsy.” This innovative approach can not only enhance our understanding of the biological mechanisms underlying dissemination and metastasis but also holds promise in developing improved biomarkers for the more accurate detection, analysis, and treatment of systemic cancer.

Despite these potential benefits, the detection of CTCs remains a challenging task. Their concentration in the bloodstream is extremely low, and they lack specific markers that are unique to cancer cells, limiting their utility as a diagnostic tool. In response to these challenges, numerous platforms and techniques have been developed to enrich, detect, and isolate CTCs from blood samples, aiming to improve their detection sensitivity and reliability.

The aim of this narrative review is to offer the reader a solid base to understand the current state of CTC analysis and its clinical significance in patients with epithelial cancers. By examining the advancements in CTC detection methods, exploring their clinical applications, and addressing the challenges ahead, we hope to provide a timely overview of the current understanding of CTCs and their potential implications for clinical practice.

## Principles and methods for CTC detection and enrichment

**Figure 1:**
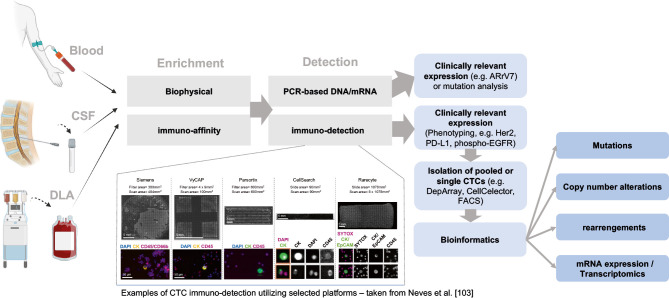
Overview of sample types processed wit CTC technologies and possibilities for subsequent analysis (designed with BioRender).

Traditionally, two main approaches have been employed to detect epithelial CTCs in blood samples: antibody-based immune-staining and PCR-based molecular detection methods (Figure 1). In immuno-staining, antibodies are utilized to identify epithelial antigens on CTCs. In PCR-based methods, the focus is on detecting cancer-specific DNA mutations, DNA hypermethylation, or cancer-associated mRNA transcripts, which are more commonly used [3–10]. Additionally, fluorescence in situ hybridization (FISH) has emerged as a technique for detecting cancer-specific aneusomy in CTCs [11], and mRNA hybridization with padlock probes has been employed to detect cancer-specific mRNA transcripts [12].

The origins of CTC detection through immuno-staining can be traced back to protocols developed in the late 1980s for detecting disseminated cancer cells in the bone marrow and lymph nodes, which were subsequently adapted for blood samples. Early techniques, such as cytology and flow-cytometry, were able to detect one tumor cell in 100 normal blood cells, while more sensitive immune-cytochemistry methods achieved a detection limit of one tumor cell in 10^5 blood cells [13]. Another variant of CTC immuno-detection evolved the Epispot assay, which represents an in vitro functional assay relying on immunofluorescence detection of secreted proteins markers derived from viable cancer cells in short-term culture [14]. Currently, multi-marker immunofluorescence staining is the most common approach to identifying epithelial tumor cells. This involves the application of antibodies against cytokeratins, which are characteristic intermediate filaments of epithelial cells, or against other epithelial or malignancy-associated markers like EpCAM, ERBB2, MUC-1, EGFR (including phosphorylated states), PSMA, or VAR2 [15–21]. The use of antibodies against CD45 as an exclusion marker to label leukocytes has become standard in most current assays. Additionally, nucleated acid dye such as DAPI is used to identify intact nucleated cells. Such immunostainings are implemented in most semi-automated platforms for CTC detection, including CellSearch [22], Epic Sciences [23], and RareCyte [24]. It should be noted that although the EpCAM+/CK+/CD45-/DAPI+ phenotype for CTC identification used in the CellSearch platform is generally accepted preferably there would be an universally defined CTC phenotype. This however is quite complex as different technologies employ divergent antibody clones with different fluorophores which are detected by different microscope platforms, complicating the comparison and standardization of results across various platforms.

In comparison to immuno-detection, PCR-based molecular CTC detection achieved already in the 1990s a remarkable sensitivity. PCR assays could detect even a single malignant cell among up to 10^7 normal cells [13]. In the context of epithelial cancers, CK mRNA has frequently been utilized for CTC detection [9]. The AdnaTest® is perhaps the most widely used commercial kit for CTC detection through mRNA profiling. It employs a multiplex reverse-transcription (RT-)PCR approach targeting a panel of transcripts as surrogate for the presence of CTCs in a cell suspension obtained from blood after immuno-magnetic enrichment of epithelial cancer cells [25]. A more recent development in detecting CTC-derived nucleic acids is the application of digital PCR, which is commonly 1–2 logs more sensitive than standard PCRs [26, 27]. However, while PCR-based CTC detection may provide clinically valuable information, it does not facilitate the isolation of pure CTCs for subsequent downstream molecular analysis as demonstrated for immuno-detection protocols [28, 29]. Another potential limitation of PCR-based detection assays involves the analysis of mesenchymal antigen expression, as it remains ambiguous whether the detected signal emanates from the tumor cells themselves or originates from the variable background presence of white blood cells.

Given the typically low concentration of CTCs in blood, ranging commonly between 1 and 50 CTCs in a positive sample [2], effective enrichment techniques are essential for their detection and analysis (Figure 1). A standard 10 mL peripheral blood sample contains billions of red blood cells, thrombocytes, and approximately 4.5–6x10^7 leukocytes, making some form of enrichment necessary even for sensitive molecular assays. One of the earliest approaches for enriching epithelial cells from blood samples was density gradient centrifugation, which enriched epithelial cells together with the mononuclear cell fraction due to a similar density of around 1.077 mg/L [30]. Another early biophysical enrichment approach used until today employs filtration techniques exploiting size and deformability differences between blood cells and CTCs [31]. However, it is important to recognize that the lack of specificity in biophysical separation methods can be a drawback to the efficacy of enrichment, as the biophysical characteristics of CTCs and leukocytes often overlap to some extent. For instance, filtration devices with eight μm pore sizes, such as ISET (Isolation by Size of Epithelial Tumor Cells), would inadvertently capture leukocytes while losing smaller CTCs [2].

More specific enrichment methods emerged utilizing immuno-magnetic techniques, either through positive selection targeting epithelial cell-surface antigens or negative selection targeting leukocyte-specific antigens (e. g., CD45). Despite its high specificity, the absence of common cancer-specific antigens and the potential dynamic expression of surface proteins in cancer cells poses a challenge for this approach. This can be exemplified by the EpCAM, the most widely used surface protein for positive selection, which is also utilized in the current “gold standard” for CTC detection, the FDA-cleared CellSearch system. EpCAM is commonly expressed in most carcinomas, providing specificity for epithelial cells, although it is also found in embryonic stem cells and limited subsets of adult stem and progenitor cells [32]. However, EpCAM is not universally expressed in all cancer types. Its expression can be heterogenous, absent, or downregulated in certain cancers, such as squamous cell carcinomas, and especially if cancer cells undergo epithelial to mesenchymal transition (EMT) [33–32]. Consequently, EpCAM-based CTC detection will miss CTCs. However, several studies provided evidence for the important role of EpCAM in metastasis across different cancer types, suggesting that EpCAM identifies relevant CTC populations.

A significant advancement in the field of CTC enrichment was the introduction of microfluidic chips offering versatility and also automation potential. The pioneering publication by Nagrath et al. was a breakthrough for microfluidic CTC enrichment [34]. This microfluidic chip utilized immunoaffinity capture via antibody-coated posts. Since then, various microfluidic platforms have been developed, capitalizing on the physical and biological properties of CTCs in comparison to normal blood cells [35, 36]. These biofluidic technologies, which include immunoaffinity capture, size-based filtration, deformability-based sorting, vortex-sorting, or a combination of these approaches, aim to exploit distinct properties to effectively enrich CTCs while minimizing interference from other blood components. The utilization of microfluidic chips has allowed for precise manipulation and control of fluid flow within small-scale channels, facilitating a more precise capture of CTCs [37]. Immunoaffinity capture involves chip surfaces functionalized with specific antibodies to selectively bind and capture CTCs based on their surface markers (e. g., EpCAM). Size-based filtration relies on the size difference between CTCs and other blood cells to separate and retain CTCs within specific regions of the chip. Deformability-based sorting takes advantage of the varying deformability of CTCs compared to normal blood cells, allowing for their separation based on differences in mechanical properties. Vortex-sorting utilizes hydrodynamic forces and fluid dynamics within microfluidic channels to concentrate and isolate CTCs in specific regions of the chip’s microfluidic sorting channel [35–37]. By harnessing these biofluidic technologies, the efficiency and sensitivity of CTC enrichment can be enhanced, enabling downstream analysis and characterization of these cells. As for conventional biophysical separation, potential drawbacks arise from overlapping characteristics of CTCs and normal blood cells [2]. However, by continuously refining and combing these enrichment methods, future technologic developments aim to overcome the challenges posed by the low abundance and heterogeneity of CTCs in blood samples. For the time being, it is important to acknowledge that currently, no enrichment method achieves 100 % efficiency, and therefore, there is always a degree of cell loss that cannot be precisely quantified for each individual sample.

## Clinical validation of CTCs

Although over 40 CTC assays based on the principles mentioned above have been published [38] [39], only a handful have been consistently reported as endpoints or pharmacodynamic markers in clinical trials. Among them, the CellSearch system, remains the sole system FDA-cleared for the detection and monitoring of CTCs in patients with metastatic breast, prostate, and colorectal cancers. Due to its significant impact, this section primarily focuses on the data generated using the CellSearch system, which has played a crucial role in establishing reliable clinical evidence that conclusively demonstrates the prognostic impact of CTCs. CellSearch is a system that utilizes ferrofluids and EpCAM-targeting monoclonal antibodies to capture epithelial cells from 7.5 mL of blood [22]. The captured cells are then stained with Phycoerythrin (PE)-conjugated antibodies for cytokeratins (CKs) as well as an Allophycocyanin (APC)-conjugated antibody for CD45. Additionally, the enriched cells are stained with DAPI to ensure the detection of intact nucleated cells. Images of the processed samples are acquired by a computer-controlled fluorescence microscope. Notably, the CellSearch-Magnest device holds magnetically labeled cells (EpCAM+) in place, preventing cell loss during processing. A trained operator screens an image gallery of DAPI+ and CK+ objects generated by a computer algorithm to identify CTCs, which are defined as EpCAM+/CK+/CD45- objects with a cellular shape, size of at least 4x4 µm and a DAPI+ nucleus positioned at least 50 % within the cytoplasm [40]. Several prospective multicenter studies established CTCs according to this definition as prognostically relevant. A count of ≥5 CTCs in metastatic breast and metastatic prostate cancer and ≥3 CTCs in metastatic colorectal cancer (CRC), respectively, has been strongly correlated with poor survival [41–44]. Similar prognostic significance has been observed in non-small cell lung cancer [45], small cell lung cancer [46], gastric cancer [47], pancreatic and peri-ampullary cancer [48], and head and neck cancer [49]. Moreover, in advanced prostate cancer, the combination of elevated serum lactate dehydrogenase (LDH), a biomarker indicating aggressive malignancy, at week 12 of treatment, along with CellSearch CTC counts, fulfills all four Prentice criteria, establishing CTC counts as an individual patient-level surrogate for survival [50]. In patients with ≥5 CTCs in 7.5 mL of blood prior to therapy, a ≥30 % decrease in CTC counts as early as week four has been associated with longer survival [51]. A recent re-analysis, utilizing individual patient data from five prospective randomized phase III trials involving >6,000 patients, identified negative CTC CellSearch test (CTC0) as a readily identifiable and clinically significant endpoint. Since this endpoint can be determined shortly after initiating treatment indicating therapy benefit, the use of CTC0 as a response endpoint has been recommended for early-phase clinical trials [52]. However, the predictive value of CTC enumeration before treatment initiation, i. e., providing information on the probability of response to a specific therapy, has yet to be established in prostate cancer. This limitation currently hinders the routine clinical use of CTC enumeration, despite its well-documented strong prognostic value.

## The clinical value of CTC detection in non-metastatic cancer

Most data are available for studies with the CellSearch system, and localized breast cancer is the so far best-investigated cancer for the presence of CTCs. It has been shown to be an independent prognostic factor for disease-free survival and overall survival. CTC counts have also been associated with relapse-free survival and response to radiotherapy. In the neoadjuvant setting, pre-chemotherapy CTC counts have been linked to worse overall survival and distant disease-free survival, with higher CTC counts correlating with increased mortality. Strikingly, CTCs can also be detected in peripheral blood samples in the minimal residual disease (MRD) situation after complete removal of the primary tumor [53–54], after all cycles of neo-/adjuvant therapy were completed [55–56] and even two years after completion of the adjuvant therapy [57]. Apparently, micro-metastatic cancer cells re-enter the bloodstream and become then detectable as rare CTCs. The observation that these MRD-CTCs can predict recurrences suggests that they reflect active MRD. Interestingly, CellSearch detected CTCs in early breast cancer were used for therapeutic decision making in the EORTC 90091–10093 BIG 1–12 Treat CTC trial [58]. This randomized trial aimed to determine whether trastuzumab reduces the detection of ≥1 CTC in high-risk, HER2 nonamplified, early breast cancer. However, the study results showed that trastuzumab did not decrease the detection rate of CTCs in this BC subtype. The trial was terminated early due to futility, as there was no significant difference in CTC detection between the trastuzumab and observation arms. Limitations of this study were the low frequency of CTCs and the fact that HER2 was not assessed on the “treated” CTCs.

As in the metastatic situation, pre-operative CTC detection has been identified as an independent prognostic marker for poor outcomes in localized colorectal cancer [59]. Limited data are available for other cancer types, such as pancreatic cancer including adenocarcinoma of pancreas (PDAC), where pre-operative detection has shown to be an independent prognostic factor for overall survival [48]. The presence of CTCs has been associated with a higher risk of relapse in non-metastatic non-small cell lung cancer patients and several other cancer types. However, studies on localized prostate cancer have yielded conflicting results [60].

The low detection rates of the CellSearch system in localized cancers suggest that this system has no role in screening and early cancer detection. Other systems, such as the filtration system known as ISET, have reported higher detection frequencies, particularly in lung cancer [61]. In a prospective multicenter cohort study, the potential of ISET CTCs as a biomarker for lung cancer screening was evaluated [61]. The study enrolled 614 participants eligible for lung cancer screening and with chronic obstructive pulmonary disease. Participants underwent three screening rounds over one-year intervals, including low-dose chest CT and blood tests for CTC detection. However, the results showed that CTC detection using ISET was not suitable for lung cancer screening, with a sensitivity of only 26 % and an inability to predict lung cancer or extrapulmonary cancer development. These findings indicated that CTC detection using ISET may not be a viable option for lung cancer screening. A plausible explanation for this inconclusive study result may reside in the dedifferentiated character of NSCLC (Non-Small Cell Lung Cancer) CTCs, coupled with the issue of overlapping cell sizes between CTCs and normal white blood cells [62].

## The clinical utility of CTC enumeration in metastatic cancer

While the clinical validity of CellSearch-CTCs has been remarkably established and response to therapy can be clearly monitored via CTCs, this biomarker could not be translated into clinical routine yet. Obviously, the design of studies demonstrating clinical utility, thus a survival benefit from the CTC analysis, can be complex, especially in the context of constantly evolving and changing standards of care. This might be one reason why relatively few prospective randomized clinical trials have been conducted so far. The first published clinical trial investigating the predictive potential of CellSearch-CTCs was the SWOG-S0500 trial carried out in MBC [63]. The study included 595 patients with metastatic breast cancer and tested whether a CTC-driven change of therapy can improve overall or progression-free survival. The trial was negative and found no survival benefit but confirmed the strong prognostic significance of CTC enumeration by CellSearch. Challenges discussed included the timing of CTC enumeration and the need for additional markers to predict response to alternative therapies [2]. The CirCe01 trial was also carried out in MBC and aimed to evaluate the clinical utility of CTC-based treatment decisions beyond the third line of therapy [64]. The trial enrolled MBC patients after two lines of chemotherapy, and those with ≥5 CTCs per 7.5 mL were randomized between the CTC-driven and standard arms. In the CTC arm, changes in CTC count were assessed during each line of chemotherapy, and patients with CTC levels indicating early tumor progression had to switch to the next line of chemotherapy. However, due to limited accrual and compliance, the trial could not demonstrate the clinical utility of CTC monitoring. Overall survival (OS) was not different between the CTC-driven and standard arms, but subgroup analysis showed that patients who switched chemotherapy based on CTC response experienced longer survival compared to those who did not.

The recently published STIC CTC trial, a randomized clinical trial involving 755 women with hormone receptor-positive, Her2-negative MBC, could demonstrate the clinical utility of CTC enumeration [65]. Here, the efficacy of the CTC-driven treatment choice was compared to a clinician-driven choice for first-line treatment. The trial demonstrated that CTC counts can guide the selection between chemotherapy and endocrine therapy. The CTC arm, where treatment was based on CTC count (≥5 CTCs/7.5 mL for chemotherapy; <5 CTCs/7.5 mL for endocrine therapy), showed a median progression-free survival of 15.5 months compared to 13.9 months in the standard arm. However, despite its promising results, as the authors noticed, a significant limitation of the STIC trial was that it was designed before CDK4/6 inhibitors became the standard of care [65]. As a result, the study did not specifically address the conundrum between endocrine therapy and chemotherapy in patients who progress on a CDK4/6 inhibitor used as first-line or adjuvant therapy. This decision-making challenge remains unresolved in clinical practice, emphasizing the need for further research and integration of biomarkers like CTC count to guide treatment choices in this context [60].

In metastatic colorectal cancer, a randomized phase III multicenter study (VISNÚ-1) aimed to investigate the role of CTC counts to select patients for a more intense therapy with FOLFOXIRI-bevacizumab in metastatic colorectal cancer versus FOLFOX-bevacizumab [66]. The study included 349 previously untreated patients with unresectable metastatic colorectal carcinoma and ≥ 3 CTCs per 7.5 mL blood. The results showed that FOLFOXIRI-bevacizumab significantly improved progression-free survival (PFS) compared to FOLFOX-bevacizumab in this patient population, with a median PFS of 12.4 months versus 9.3 months, respectively. Grade ≥3 adverse events were more common with FOLFOXIRI-bevacizumab. The findings suggest that CTC count may be a valuable non-invasive biomarker for selecting patients who could benefit from intensive first-line therapy. As the authors discussed, subsequent studies would be of interest to investigate the escalation or de-escalation of therapy according to CTC counts after therapy initiation. One critique of this study centered around its focus on the primary endpoint of PFS while comparing a triplet chemotherapy backbone to a doublet backbone [67]. It was acknowledged that a more significant endpoint would have been OS or the duration of time until the occurrence of a second progression following the administration of a second doublet treatment for those individuals who initially received a doublet treatment in the first line.

## The clinical utility of CTC profiling in metastatic cancer

The level of information provided by CTC numbers is apparently limited, especially in the context of targeted therapies or immunotherapies. To augment the depth of information garnered, the CellSearch system introduces a fourth fluorescence channel, permitting additional antibody staining – such as evaluating the expression of a therapeutic target on CTCs. This methodology was employed in the phase 3 DETECT III study, which proactively addressed intra-patient heterogeneity and sought to pinpoint initially HER2-negative MBC (metastatic breast cancer) patients who exhibited HER2-expressing CTCs, positing them as potential beneficiaries of anti-HER2 treatment [68]. A foundational study by Georgoulias et al. underscored the significance of HER2 expression on CTCs in HER2-negative MBC [69]. Through employing CellSearch in the DETECT III study, 105 patients manifesting positive HER2 CTCs were identified and subsequently randomized to assess the efficacy of the HER2-targeted therapy lapatinib in comparison to standard therapy. Notably, while CTC clearance at the first follow-up visit was not significantly disparate between the treatment arms at any point, it was significantly correlated with enhanced OS, presenting 42.4 versus 14.1 months (p=0.002). Besides, this study demonstrated that lapatinib had a positive impact on OS in this patient population. Patients in the lapatinib arm showed a significantly improved OS compared to those in the standard therapy arm (20.5 versus 9.1 months, p=0.009), as evidenced by hazard ratios of 0.54 (95 % CI 0.34–0.86; p = 0.008) and 0.53 (95 % CI, 0.33–0.86; p = 0.010). Although the primary endpoint of CTC clearance rate did not differ significantly between the two arms, the improved OS observed in the lapatinib arm highlights the potential clinical relevance of HER2-positive CTCs as a biomarker for predicting treatment response and clinical benefit in patients with initially HER2-negative MBC [68]. Despite the intriguing data, this study revealed an important drawback of CellSearch CTC-based diagnostics in HER2-negative breast cancer and pointed to an important aspect in general: to randomize the 105 CTC HER2-positive patients 1933 MBC patients were screened, and only 1217 out of them (63.0 %) had ≥1 CTC per 7.5 ml blood and ≥5 CTCs were detected in 735 patients (38.0 %; median 8 CTCs) [70]. This renders a large patient group as not informative at all or providing only limited, potentially unreliable diagnostic information. The full data of this landmark study are expected to be published later in 2023. The DETECT III has been, so far, the only large study in which molecular phenotyping of CTCs using the CellSearch system guided a therapeutic decision.

An interesting development for CTC-based therapy decisions emerged in metastatic castration-resistant prostate cancer (mCRPC). Here, the androgen receptor splice variant-7 (AR-V7) has garnered significant attention as a biomarker for therapeutic decision-making since elevated AR-V7 expression has been associated with resistance to androgen receptor signaling inhibitors (ARSi). Initially, using the Adna-Test, it was established that AR-V7 in CTCs is associated with resistance to ARSi [71–72]. More recently, the automated image-based Epic Sciences “no cell left behind” CTC detection platform provided a test enabling the immune-detection of nuclear AR-V7 in individual CTCs. After two studies indicated the clinical utility of the Epic Sciences assay in mCRPC patients undergoing treatment change to select patients for therapy with taxanes instead of ARSi [73–74] local health-insurance plans covered AR-V7 protein assay, commercialized as Oncotype DX AR-V7 Nucleus Detect test, for mCRCP patients in the U.S. [75] despite the lack to establish the predictive value for selection of taxanes vs. ARSi. Additional corroborating data came from the PROPHECY study that demonstrated for both mRNA- and protein-based CTC AR-V7 assays, similar value for selecting mCRCP patients to ARSi or taxane treatment in the context of other clinical parameters [76]. The authors stressed that the CTC AR-V7 status could explain some of the resistance to AR therapy, but AR-V7 heterogeneity within patients and over time suggests that additional strategies are needed to overcome resistance in such mCRCP patients.

In the context of clinical relevant CTC profiling, it is of interest that Angle’s Parsortix PC1 system received just recently (May 2022) FDA clearance as an in vitro diagnostic device intended to enrich CTCs from the peripheral blood of MBC patients [77]. The system employs a microfluidic chip for size- and deformability-based CTC capture. Interestingly, FDA clearance did not cover CTC-identification or analysis, but the end user will be responsible for the validation of any further downstream assay. Supporting data for the FDA clearance came from the HOMING Study, a multicenter, prospective, blinded study in healthy volunteers and MBC patients that successfully yielded CTCs for downstream evaluation [78]. CTCs were analyzed by qRT-PCR, RNA sequencing, or cytogenetic analysis of HER2 amplification by FISH. Notably, when the investigators used cytopathology to determine CTCs, they detected 49 % of MBC patients (n=194) CTC-positive, but also 10 % of the healthy volunteers (n=192). This is, however, not a surprising result and relates to the more subjective cytopathology without any immuno-phenotyping. Unfortunately, the study did not compare to a CTC-detection standard, such as the CellSearch system, which is a clear limitation. However, this investigation and this particular FDA clearance pave the way for clinically useful and comprehensive CTC-profiling. In principle, as reviewed elsewhere [79–81], technically exciting possibilities exist to precisely isolate individual CTCs and perform diagnostic tests on the genomic, transcriptomic, epigenomic, and proteomic level (Figure 1).

**Table 1:** Overview on studies investigating CSF for tumor cells with CTC-detection methods

**Author**	**Year**	**CTC-assay**	**molecular CTC analysis**	**Cancer type**	**patients with LM (n)**	**Main findings**	**Reference**
Patel et al.	2011	CellSearch	no	MBC	5	first application of CS on CSF, monitoring of response to therapy	[104]
Le Rhun et al.	2012	CellSearch	no	MBC	8	range 1–10,500/5mL CSF	[105]
Le Rhun et al.	2013	CellSearch CMC	no	Melanoma	2	range 5–1,090 CMC/5mL CSF	[106]
Magbanua et al.	2013	IE/FACS (developed for CTCs in blood)	yes, aCGH	MBC	15	genome profiling in 13/15 patients, typical MBC profiles with subcloncal changes compared to biopsy tissue	[107]
Nayak et al.	2013	CellSearch	no	MBC, lung, others	16	Median CSF CTC/mL: 19.3; sensitivity of 100 % as compared with 66.7 % for conventional cytology and 73.3 % for MRI, one patient false positive	[108]
Tu et al.	2015	CellSearch	no	lung	18	Median CSF CTCs/5 mL: 785; sensitivity of 77.8 %, compared with 44.4 % for conventional cytology	[109]
Jiang et al.	2017	CellSearch	yes, NGS pane of 416 genes	Lung (NSCLC)	21	Sensitivity of 95.2 % versus 57.1 % cytology, and versus cytology + MRI (90.5 %), Genetic profiles of CSF CTCs highly concordant with molecular mutations identified in the primary tumor (17/19, 89.5 %); EGFR T790M detected in 7/9 patients with extracranial lesions, but only in 1/14 CSF CTC samples.	[110]
Lin et al.	2017	CellSearch	no	MBC, Lung, others	30	Sensitivity of 93 %, specificity of 95 %, positive predictive value 90 %, and negative predictive value 97 %	[111]
Torre et al.	2020	CellSearch	no	MBC, Lung, others	20	Sensitivity of 88.9 % and specificity of 100 % for detecting LM – threshold of 1 CTC/mL of CSF	[112]
Malani et al.	2020	CellSearch	no	Her2+ MBC	15	Median CSF CTC/3mL: 22; cytology in 40 % and CSF CTCs identified in 87 %. HER2-expression of CTCs assessed, under therapy; 75 % with of HER2 positivity in CSF	[113]
Darlix et al.	2022	CellSearch	yes, HER2 phenotyping	MBC	40	Median CSF CTC: 5824; sensitivity of100 % and specificity of 77 % for LM diagnosis. HER2+ CSF CTCs detected 41 % of patients with HER2-BC	[114]
Wooster et al.	2022	CNinside	yes, HER2 & ER phenotyping	MBC	10	sensitivity OF 100 % and specificity of 83 % for LM; concordance of ER and HER2 status between CSF-TCs and metastatic biopsy: 60 % and 75 %, respectively	[115]

MBC: metastatic breast cancer; CS: CellSearch; CSF: cerebrospinal fluid; aCGH: array comparative genomic hybridization; NGS: next generation sequencing; MRI: magnetic resonance imaging; ER: estrogen receptor

## Application of CTC-technologies to cerebrospinal fluid

Finally, an intriguing advancement in the CTC field is the application of highly sensitive CTC-detection platforms to investigate the presence and relevance of tumor cells within cerebrospinal fluid (CSF). Leptomeningeal metastasis (LM) occurs in approximately two to eight percent of patients with solid tumors. The current diagnostic methods for LM rely on clinical symptoms and the presence of contrast enhancement in the leptomeninges on brain and/or spine magnetic resonance imaging (MRI). However, MRI has limited sensitivity (around 76 %) and specificity (around 77 %) for accurately diagnosing LM. In cases where MRI scans are inconclusive, a lumbar puncture (LP) is performed to obtain CSF. The sensitivity of CSF cytology, which is used to detect cancer cells in the CSF, is also relatively low. It ranges from 44 to 67 % upon the first LP and increases to 84 to 91 % upon repeated sampling [82]. The enrichment and identification of epithelial CSF CTCs offer a new avenue to improve LM diagnostics o the central nervous system (CNS) and assessing the effectiveness of therapies targeting CNS metastasis. Moreover, this approach offers significant prospects for unraveling underlying mechanisms of CNS metastasis. Several studies have provided evidence of CTCs’ existence in the CSF of patients with diverse malignancies, including breast, lung, and melanoma (**Table 1**). When compared, CSF CTC analysis outperforms not only cytology and MRI but also CSF ctDNA analysis in terms of specificity and sensitivity. This has been corroborated in a recent meta-analysis [83]. Besides more precise clinical diagnostics for presence of LM, the analysis of CSF-derived CTCs can provide valuable information on tumor heterogeneity, genomic alterations, and treatment response specific to the CNS compartment. Furthermore, the monitoring of CTCs in CSF may offer insights into the early detection of CNS metastases and the development of targeted therapeutic strategies. Although the field is still evolving, the exploration of CTCs in spinal fluid presents an exciting development for advancing our understanding of CNS involvement in cancer and holds promise for improving patient management in the future.

## Outlook and current challenges and

CTCs have undergone extensive examination over the last two decades, consistently nearing, but not fully integrating into, clinical use despite numerous studies. To date, the translational impact of CTCs into meaningful clinical applications has been restricted. It is anticipated that CTC assays may find a place in the routine clinical management of certain cancers that exhibit more robust CTC detection frequencies and counts, such as mCRPC or small-cell lung cancer. Applications that identify the expression of therapeutic targets on CTCs highlight the utility of this liquid biopsy analyte. Specific instances, such as the application of the ARv7 CTC assays for mCRPC in selected U.S. states (as discussed above), serve as demonstrations of gradual integration, with potential for broader adoption in the future. Looking ahead, developments seem to be tentatively exploring the combination of different liquid biomarkers. For instance, the commercially available defineMBC assay, which combines phenotypic and genomic data derived from CTCs with ctDNA analysis, exemplifies this developmental trajectory. It will be crucial to observe how these and similar applications evolve in a clinical setting, ensuring that they undergo rigorous testing and validation in diverse patient cohorts.

However, major challenges must be addressed for the more widespread clinical use of CTC applications. A significant limitation is their extremely low concentration in the entirety of a patient’s blood volume, resulting in low CTC detection frequency and number. Given the Poisson distribution and the estimated quantity of cancer cells ranging from five to 250,000 in the total five-liter blood volume [2], the probability of capturing them in a 10 mL blood sample is low. To overcome this challenge, the concept of high-blood volume analysis has emerged, particularly when molecular profiling of CTCs is desired. High-blood volume analysis can be enabled by diagnostic leukapheresis (DLA) [84], a clinically safe method allowing to screen around 2.5-liter blood for CTCs. Leukapheresis is a clinical routine procedure that collects mononuclear cells (MNCs) from the blood by continuous density-based cell separation. Since epithelial cells have similar densities as the targeted MNCs (1.055–1.08 g/mL), CTCs become co-collected during the process and increase the CTC-detection frequency and yield, which has been convincingly shown by several groups across different cancer types [85–92]. The quality of the harvested cancer cells is high, as demonstrated by the expansion and cultivation of viable DLA-derived CTCs [86, 88–90, 92]. However, a technical challenge remains in the processing of whole DLA products containing around 2 billion leukocytes. Notably, several technical solutions are under development to tackle this problem in DLA [93, 94] but also develop alternative strategies, such as indwelling functionalized catheters to catch CTCs [95] and even wearable devices [96]. Likely, in the not-so-distant future, integrated high-blood volume solutions will become available to not only allow CTC-based profiling in almost every metastatic cancer patient but also to significantly increase CTC-detection sensitivity in organ-confined cancer patients.

The challenges of observer-based CTC detection subjectivity and the complexity of subsequent molecular CTC analysis (from “simple” phenotypic to single cell omics) call for innovative solutions. Machine learning and AI-based imaging analysis offer promising advancements in this context [97–99]. These technologies utilize pattern recognition algorithms and deep learning models to enhance the accuracy and efficiency of CTC detection and analysis. The Epic Sciences platform [100], along with similar new approaches for other platforms such as CellSearch [97, 99], exemplifies the application of machine learning in CTC analysis. By training machine learning algorithms to identify distinctive morphological and molecular features of CTCs, these technologies enable improved detection and classification with high sensitivity. This automated and standardized approach has the potential to revolutionize CTC analysis, particularly in predicting therapeutic outcomes, and facilitate their clinical implementation.

In fact, standardizing CTC technologies and the subsequent molecular analysis poses another significant challenge, and it is imperative to ensure quality control throughout the entire process of CTC analysis, from pre-analytic steps to data analysis. This urgency arises from the growing incorporation of CTC analysis in clinical trials and the potential integration of CTC analysis into routine clinical practice [101]. However, standardization efforts face obstacles due to the diverse detection platforms that employ varying detection methods and even definitions of CTCs. Additionally, there is a lack of standardized systemic external quality assurance programs specifically designed for CTC detection, which poses a significant challenge. Therefore, the European CANCER-ID consortium has undertaken crucial steps to develop guidelines and define minimal performance qualification requirements [102, 103] prior to the clinical validation and integration of CTC assays in clinical trials. These efforts aim to ensure rigorous quality control and enhance the accuracy, reproducibility, and robust implementation of CTC analysis in routine clinical practice. Building upon these initiatives, the European Liquid Biopsy Society (ELBS) is actively engaged in advancing the field further by promoting scientific collaborations, facilitating knowledge exchange, and fostering the development of standardized protocols and methodologies for CTC analysis. Through these collective endeavors, the goal is to elevate the scientific rigor and reliability of CTC-based diagnostics, ultimately translating into improved patient care, treatment selection, and clinical outcomes.
